# School Bullying, Bystander Behavior, and Mental Health among Adolescents: The Mediating Roles of Self-Efficacy and Coping Styles

**DOI:** 10.3390/healthcare12171738

**Published:** 2024-08-31

**Authors:** Xu Wang, Leiyu Shi, Yunzhi Ding, Bowen Liu, Hongbao Chen, Wei Zhou, Renjie Yu, Peiyun Zhang, Xin Huang, Yong Yang, Zhijun Wu

**Affiliations:** 1 Department of Health Policy and Management, Johns Hopkins Bloomberg School of Public Health, Johns Hopkins University, Baltimore, MD 21205, USA; rayman.wangxu@jhu.edu (X.W.);; 2 Vanke School of Public Health, Tsinghua University, Beijing 100084, China; 3 Suzhou Guangji Hospital, Suzhou 215137, China; 4 Affiliated Guangji Hospital of Soochow University, Suzhou 215137, China

**Keywords:** bullying, positive bystander, negative bystander, mental health, mediating role

## Abstract

While numerous studies have revealed the impact of different bullying behaviors, such as victimization and perpetration, on the psychological development of adolescents, the exploration of the correlates of positive/negative bystander behaviors and their potential underlying mechanisms remains scarce in China. The present study aims to compare the relationships between mental health and positive versus negative bystander behavior and to clarify whether self-efficacy and coping styles mediate the relationships between mental health and bullying dynamics. The current study was conducted on 11,734 students from 18 secondary schools in Suzhou, China (*Mean*_age_ = 15.00, *SD*_age_ = 1.47; 53.8% boys). The information on bullying victimization, perpetration, positive/negative bystander behaviors, as well as self-efficacy, coping styles and mental health variables (including depression, anxiety, sleep disturbance, suicide risk), were collected. Negative bystander behavior was positively associated with mental health problems, while positive bystander behavior was negatively associated with these factors. Also, further analysis showed that coping styles and self-efficacy mediated the relationship between different bullying behaviors and mental health outcomes. The results highlighted the comparison of the correlates of positive and negative bystander behaviors, which were comparably crucial to those of victims and perpetrators for prevention and intervention efforts. Promoting adaptive coping styles and self-efficacy to buffer the deleterious psychological consequences of bullying behavior in adolescents was also important.

## 1. Introduction

In the past decades, there has been a growing awareness of the mental health of adolescents, with an increased emphasis on factors beyond academic success. Almost one in seven adolescents globally live with a mental health condition [1], within which symptoms of depression and anxiety make up about 43% [2]. These conditions may be associated with numerous adverse outcomes, such as impaired academic performance, deteriorating relationships with family and peers, clinical outcomes of physical diseases, and a higher likelihood of engaging in risky behaviors [3,4,5]. These conditions can lead to chronic health issues and challenges in forming stable careers and relationships in adulthood [6,7].

Addressing adolescent mental health necessitates identifying the key factors that contribute to mental disorders. School bullying is a widely recognized risk factor for the mental health of adolescents [8,9]. Bullying is defined as the intentional exposure to repeated negative actions from a perpetrator to a victim with a power imbalance [10]. Globally, 32% of students have experienced bullying by their peers at least once within a month [11]. A systematic review estimated the prevalence of overall bullying victimization at 22.7% and bullying perpetration at 15.7%. Additionally, the estimated prevalence of face-to-face bullying victimization was more than twice as high as that of cyberbullying victimization [8,12]. Generally speaking, conventional bullying involves three distinct roles: the victim, the perpetrator, and the witness or bystander [13]. Previous studies have consistently shown that bullying victims are susceptible to mental health disorders, including anxiety [14,15], depression [16], and suicidal intentions [17,18,19]. Bullying victimization also negatively affects body image, potentially leading to conditions such as muscle dysmorphic disorders, which are linked to elevated levels of psychopathology [20]. Contrary to common assumptions, recent research indicates that bullying perpetrators are also adversely affected by their behaviors [21,22,23,24,25,26]. The reason could be attributed to adverse childhood experiences, including exposure to violence, neglect, or inconsistent parenting [27]. Additionally, deficiencies in self-regulation and higher impulsivity may make perpetrators suffer from poor peer relationships, which can exacerbate feelings of loneliness, rejection, and self-doubt [28].

However, in the context of bullying, there is an important yet often overlooked role—the bystander. Over 70% of students once reported witnessing bullying as bystanders [29]. Generally, bystanders can be divided into two primary categories: positive and negative bystanders. Positive bystanders were defined as individuals who intervene or seek to address instances of bullying and took actions confronting the aggressor or seeking assistance from authority figures, as well as indirect actions like offering support to the victim after the incident [30,31]. Negative bystanders were characterized by a reluctance or failure to intervene in bullying situations [32]. Prior research has identified distinct pathways and psychological motivations underlying these two bystander types [33]. Highly empathetic students tend to be more inclined to be positive bystanders in an environment with a good student–school climate and connectedness [34,35]. Psychological mechanisms including the diffusion of responsibility and moral disengagement discourage bystander intervention [36,37]. A minority of studies have found that the bullying behaviors can also adversely affect the mental well-being of bystanders [38,39,40,41]. However, the impact of bystander behavior on psychological health outcomes remains understudied, particularly in the context of Chinese adolescents. Moreover, distinctions between different bystander roles and their respective mental health implications have not been thoroughly explored. Therefore, based on differing psychological mechanisms and roles, we hypothesize that positive and negative bystanders will show distinct associations with mental health outcomes.

Having recognized the correlation between mental health and various roles in bullying, it is essential to identify and understand the cognitive mechanisms that explain this relationship [9]. Understanding why victims, perpetrators, and bystanders experience compromised psychological health outcomes is necessary for effective prevention and intervention. According to the Transactional Model of Stress and Coping proposed by Lazarus and Folkman [42], stress arises when individuals perceive a situation as exceeding their resources to cope. Coping strategies and self-efficacy as psychological resources play an important role in how individuals manage stress [43]. Self-efficacy is defined as an individual’s perceived capability to execute a specific behavior and achieve a desired goal [44]. Coping styles refer to the diverse strategies individuals use to manage stressors, with relatively stable traits influencing their responses [45,46,47]. Self-efficacy influences coping styles significantly. Higher self-efficacy is linked to more adaptive coping strategies and better mental health, while lower self-efficacy often leads to maladaptive coping and increased stress [48]. Enhancing self-efficacy can improve coping and psychological well-being [49]. Both of them may influence how adolescents perceive and respond to bullying behaviors, affecting overall well-being and resilience. For example, a longitudinal study found that the coping style could mediate the association between stressful life events and mental health outcomes for young Canadians [50]. Meanwhile, self-efficacy was also a mediator of mental well-being and bullying victimization for youth [51,52]. However, limited research has investigated the mediating role of coping styles and self-efficacy in the relationship between bullying and mental health, particularly from the perspective of bystanders. Moreover, there is a scarcity of studies that examine the different types of bystanders. Therefore, based on the previous studies and research gap, the second hypothesis of this study is that self-efficacy and coping styles may play mediating roles in the relationship between bullying behavior and mental health outcomes. Moreover, the mediating paths of different roles in bullying behavior are different.

To sum up, although substantial research has examined the impact of bullying on mental health outcomes among adolescents, significant gaps remain in understanding the nuanced roles of different bystander behaviors and the cognitive mechanisms that mediate these effects. While existing studies have established that bullying victims and perpetrators experience adverse mental health outcomes, there is limited research on how subgroup bystander roles—specifically positive and negative bystanders—affect mental health differently in China. Furthermore, the role of self-efficacy and coping styles as potential mediators in these relationships remains underexplored.

In light of these considerations and existing theories, the objective of this study is to examine the relationship between bullying and mental health among Chinese adolescents, particularly focusing on the contrasts between positive and negative bystander behaviors and between victims and perpetrators. Additionally, this study aims to investigate the cognitive mechanisms that mediate the associations between bullying dynamics and mental health outcomes. Specifically, this research explores the extent to which self-efficacy and coping styles mediate the relationships between mental health indicators—such as anxiety, depression, sleep disturbance, and suicide risk—and four types of bullying behaviors within a sample of secondary school students. Consistent with previous studies, we hypothesize that these two factors may serve as potential mediators in these relationships.

## 2. Methods

### 2.1. Participants and Study Design

Data were collected using a self-report questionnaire conducted in Suzhou City, China, from December 2018 to January 2019. A cluster sampling method was used to select all 18 public secondary schools in Xiang Cheng district among six urban districts of Suzhou. Appropriate sample sizes were drawn randomly from these 18 schools. A total of 12,354 students from 18 secondary schools participated in this survey, with response rate of 83.2%. A total of 11,734 valid responses were included in the analysis after excluding 620 invalid questionnaires. Participation was voluntary, and there were no adverse consequences if individuals refused or later withdrew from the survey. Due to the vulnerable characteristics of respondents, trained school teachers led the data collection process. All students provided written consent and were assured of the confidentiality of their completed questionnaires. This study adhered to the Strengthening the Reporting of Observational Studies in Epidemiology (STROBE) reporting guideline. Ethical approval was obtained from Suzhou Guangji Hospital, Suzhou University.

### 2.2. Measures

#### 2.2.1. Demographics

Demographic information collected for each participant included age, gender, grade level, school type, ethnic group, residence type, whether they were the only child in their families, and the educational levels of participants’ parents.

#### 2.2.2. Exposure Variables

Bullying victimization was assessed through a single question: “During this academic year, how often have you been bullied at school”? Participants had four response options for each question: “never”, “sometimes” (once or twice a month), “often” (once or twice a week), or “every day” [53]. These options were assigned scores of 0, 1, 2, and 3, respectively.

Bullying perpetration was gauged using the question: “During this academic year, how often have you bullied others at school”? Participants had four response options for each question: “never”, “sometimes” (once or twice a month), “often” (once or twice a week), or “every day” [54]. These options were assigned scores of 0, 1, 2, and 3, respectively.

Positive bystander behavior was measured with the question: “If you witness bullying at school, what would you do? Participants had four multiple choices: Didn’t see or ignored it; Would try to stop the bullying or help the victim; Would seek help from a teacher or other students” [54,55]. Scores were assigned of 0 points if they chose not to do anything, 1 point if they chose one action, and 2 points if they chose two actions.

Negative bystander behavior was measured with the question: “If you witnessed bullying at school, what would you do? Participants also had four multiple choices: Didn’t see or ignored it; Watched from the sidelines; Found it interesting and joined in” [54,55]. 0 points if they chose not to do anything, 1 point if they chose one action, and 2 points if they chose two actions, respectively.

#### 2.2.3. Mediating Variables

Self-efficacy: The Chinese version of the General Self-Efficacy scale (GSE) was used to assess individuals’ self-efficacy in dealing with life difficulties in the last year [56]. It includes 10 items, with responses on a 4-point Likert scale from 1 (not at all true) to 4 (exactly true). Scores range from 10 to 40, indicating higher self-efficacy for better adaptation to challenges. GSE scale was validated in Chinese [57], and Cronbach’s alpha in this sample was 0.9.

Positive and negative coping styles: The Trait Coping Style Questionnaire (TCSQ) was employed to assess positive and negative coping styles among the adolescents, consisting of 20 items with 10 items dedicated to each sub-scale [58,59]. An example of a positive coping statement was “I focus on the positive side and reappraise the situation”, while a negative coping example was “If I had a confrontation with someone, I might avoid communicating with that person”. Responses were rated on a 1–5 Likert scale. A higher composite score indicated a greater inclination towards adopting either the positive or negative coping style. The Cronbach’s α values were 0.85 for the positive coping style sub-scale and 0.88 for the negative coping style in this sample.

#### 2.2.4. Outcome Variables

Depression: The severity of depressive symptoms was assessed using the Patient Health Questionnaire (PHQ-9) [60]. PHQ-9 is a nine-item measure of depression based on the Diagnostic and Statistical Manual diagnostic criteria for major depressive disorder. Each item is rated on a 0 to 3 scale relating to the frequency of depressive symptoms (0 = “not at all” to 3 = “nearly every day”). Scores range from 0 to 27, with higher scores indicating a greater severity of depression. The instrument has been validated in a similar population in China and the Cronbach’s α was 0.93 [61].

Anxiety: Anxiety symptoms were measured using the Generalized Anxiety Disorder Screener (GAD-7), a tool previously validated in the general population [62]. GAD-7 is designed to assess an individual’s anxiety and has 7 items. Participants were asked to report their experiences, using a 0–3 point scale where 0 indicated “Not at all” and 3 indicated “Nearly every day”, Cronbach’s α was 0.94.

Suicide Risk: Suicide risk was assessed using the suicide module of the Mini-International Neuropsychiatric Interview [63], comprising six questions regarding participants’ suicide-related ideations and behaviors. The total scores on this module range from 0 to 31. The classification for the suicidal risk degree can be described as non-suicidal risk classes (0 score), low suicidal risk classes (1–5 score), medium suicidal risk classes (6–9 score), and high suicidal risk classes (≥10 score) [64]. It was validated by previous studies [64,65]. Cronbach’s α was 0.85.

Sleep Disturbance: The 10-item Chinese version of the Pittsburgh Sleep Quality Index was used to measure many factors related to an individual’s sleeping quality [66]. Although we examined the total score of PSQI, we selected only sleep disturbance part of the instrument due to the high number of missing values for the other parts. The scale of each item ranged from 0 to 3, with 3 indicating the highest dysfunction. The total number ranged from 0 to 30. Higher scores show poorer sleep disturbance, and a score exceeding 10 indicates significant sleep difficulties [67]. Cronbach’s alpha in this sample was 0.82.

### 2.3. Statistical Analyses

The Spearman correlation was used in SPSS version 21.0 to examine relationships among bullying involvement, self-efficacy, coping styles, and mental health indicators. Mediation analyses used Model 4 of the SPSS PROCESS macro developed by Baron and Kenny, and Hayes [68,69]. Bullying victimization, bullying perpetration, positive bystander behavior, and negative bystander behavior were treated as the independent and continuous variables [70]. Self-efficacy, positive coping styles, and negative coping styles were treated as mediators, while depression, anxiety, suicide risk, and sleep disturbance were treated as dependent and continuous variables. The mediation analyses assessed indirect effects using bias-corrected confidence intervals derived from 5000 bootstrap resamples. Age, sex, grade level (junior high school as the reference group), school type (boarding school as the reference group), one child family (yes as the reference group), education level of the father (lower than high school as the reference group), and education level of the mother (lower than high school as the reference group) were included as covariates. After standardizing the original data, the mediation models were analyzed. Listwise deletions were employed to handle the missing data of bullying-related variables, and the imputation method with the mean of items were used for mental health indicators.

## 3. Results

### 3.1. Descriptive Statistics

The analysis included 11,734 participants (*Mean*_age_ = 15.00, *SD*_age_ = 1.47). As indicated in Table 1, the majority of participants belonged to the Han ethnic group (99.7%), were male (53.8%), were from junior high school (74.5%) and day school (72.8%), resided in urban areas (56.5%), and were not the only child in their families (64.3%). The education levels of fathers (79.6%) and mothers (82.6%) for most participants were equivalent to or lower than high school. Additionally, 7.7%, 16.3%, 52.3%, and 8.2% of participants reported being victims, perpetrators, positive bystanders, and negative bystanders of bullying at their schools, respectively.

The correlation coefficients among the main variables were calculated and presented in Table 2. Negative bystander behavior was significantly positively associated with mental health indicators, whereas positive bystander behavior was not, indicating different associations for the two bystander roles. This result supports hypothesis 1. Also, involvement in bullying, negative bystander experiences, negative coping styles, anxiety, suicide risk, and sleep disturbance all exhibited positive correlations with each other.

### 3.2. Mediation Analysis

**Figure 1:**
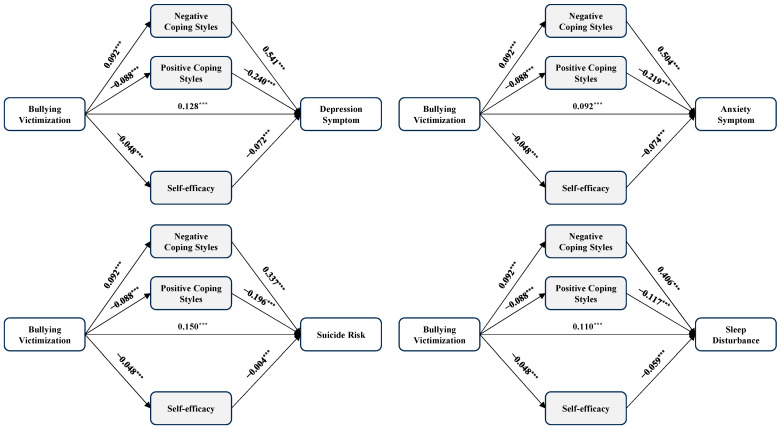
The mediation model of bullying victimization, coping styles, self-efficacy and four outcomes. Note: *n* = 10,713, *** *p* < 0.001.

**Figure 2:**
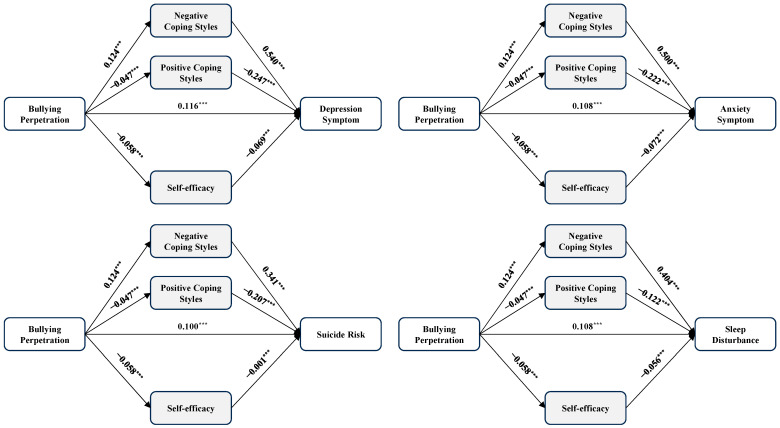
The mediation model of bullying perpetration, coping styles, self-efficacy, and four outcomes. Note: *n* = 10,713, *** *p* < 0.001.

**Figure 3:**
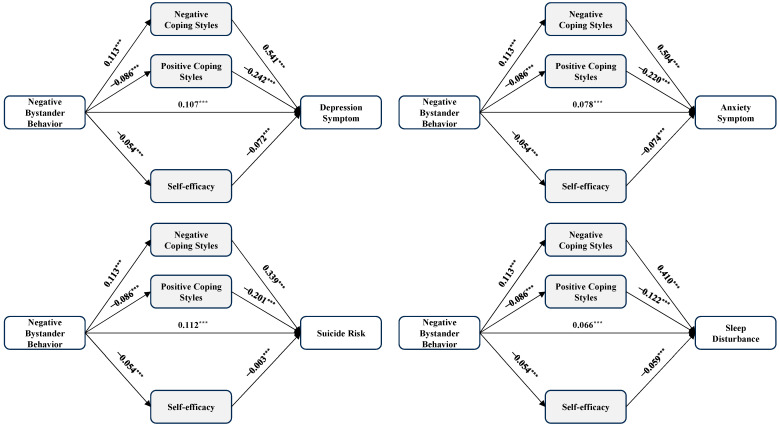
The mediation model of negative bystander behavior, coping styles, self-efficacy, and four outcomes. Note: *n* = 10,713, *** *p* < 0.001.

**Figure 4:**
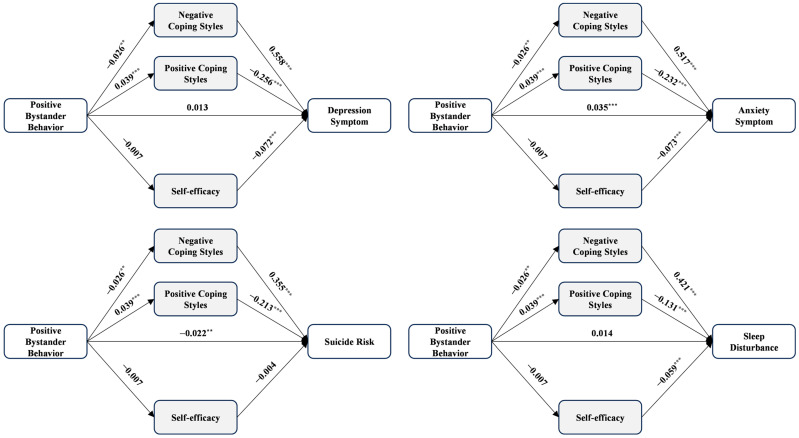
The mediation model of positive bystander behavior, coping styles, self-efficacy, and four outcomes. Note: *n* = 10,713, ** *p* < 0.01, *** *p* < 0.001.

In line with hypothesis 2, Table 3, Table 4, Table 5 and Table 6 and Figure 1, Figure 2, Figure 3 and Figure 4 presented the mediation effects of positive coping styles, negative coping styles, and self-efficacy between the four dependent variables and the four independent variables. We standardized all included variables during the mediation analyses (*n* = 10,713).

The four mediation models examining bullying victimization and mental health indicators (see Table 3 and Figure 1) revealed the following total effects: bullying victimization was positively associated with depression (*β* = 0.203, 95% CI = 0.186~0.223, *p* < 0.001), anxiety (*β* = 0.161, 95% CI = 0.144~0.182, *p* < 0.001), suicide risk (*β* = 0.198, 95% CI = 0.180~0.218, *p* < 0.001) and sleep disturbance (*β* = 0.161, 95% CI = 0.143~0.181, *p* < 0.001). The total indirect effect between bullying victimization and depression (*β* = 0.075, 95% CI = 0.064~0.086, *p* < 0.05), anxiety (*β* = 0.069, 95% CI = 0.059~0.080, *p* < 0.05), suicide risk (*β* = 0.049, 95% CI = 0.042~0.055, *p* < 0.05), and sleep disturbance (*β* = 0.057, 95% CI = 0.042~0.059, *p* < 0.05) explained 37%, 43%, 25%, and 35% of the total effect, respectively. The bootstrap results with 5000 resamples showed indirect effects of coping styles and self-efficacy, and 95% of CIs did not contain zero. Thus, coping styles and self-efficacy significantly mediated the effect of bullying victimization on depression, anxiety, suicide risk, and sleep disturbance.

The relationship between bullying perpetration and mental health indicators was examined through another four mediation models (see Table 4 and Figure 2). The total effects of bullying perpetration on depression (*β* = 0.199, 95% CI = 0.180~0.217, *p* < 0.001), anxiety (*β* = 0.185, 95% CI = 0.166~0.203, *p* < 0.001), suicide risk (*β* = 0.152, 95% CI = 0.132~0.170, *p* < 0.001), and sleep disturbance (*β* = 0.167, 95% CI = 0.148~0.186, *p* < 0.001) were observed. The total indirect effect between bullying perpetration and depression (*β* = 0.082, 95% CI = 0.072~0.093, *p* < 0.05), anxiety (*β* = 0.076, 95% CI = 0.070~0.086, *p* < 0.05), suicide risk (*β* = 0.052, 95% CI = 0.045~0.059, *p* < 0.05), and sleep disturbance (*β* = 0.059, 95% CI = 0.051~0.067, *p* < 0.05) explained 41%, 41%, 34%, and 45% of the total effect, respectively. Thus, coping styles and self-efficacy significantly mediated the effect of bullying perpetration on depression, anxiety, suicide risk, and sleep disturbance.

For negative bystander behavior (see Table 5 and Figure 3), significant total effects were observed on the key mental health outcomes: depression (*β* = 0.192, 95% CI = 0.174~0.211, *p* < 0.001), anxiety (*β* = 0.157, 95% CI = 0.139~0.176, *p* < 0.001), suicide risk (*β* = 0.167, 95% CI = 0.148~0.185, *p* < 0.001), and sleep disturbance (*β* = 0.126, 95% CI = 0.107~0.144, *p* < 0.001). These findings highlighted the pervasive impact of negative bystander behavior on various dimensions of detrimental influence. Additionally, the total indirect effect between negative bystander and depression (*β* = 0.086, 95% CI = 0.075~0.096, *p* < 0.05), anxiety (*β* = 0.080, 95% CI = 0.070~0.090, *p* < 0.05), suicide risk (*β* = 0.056, 95% CI = 0.049~0.063, *p* < 0.05), and sleep disturbance (*β* = 0.060, 95% CI = 0.052~0.068, *p* < 0.05) accounted for 45%, 51%, 34%, and 48% of the total effects, respectively. It suggested that a significant proportion of the relationship between negative bystander behavior and these mental health outcomes could be attributed to indirect pathways. These findings pointed to the critical role of coping styles and self-efficacy as mediators.

The models assessing positive bystander behavior presented a different perspective (see Table 6 and Figure 4). The total effects of positive bystander behavior on mental health outcomes were as follows: depression (*β* = −0.011, 95% CI = −0.029~0.008, *p* > 0.05), anxiety (*β* = 0.013, 95% CI = −0.006~0.031, *p* > 0.05), suicide risk (*β* = −0.039, 95% CI = −0.058~−0.020, *p* < 0.001), and sleep disturbance (*β* = −0.002, 95% CI = −0.021~0.017, *p* > 0.05). Except suicide risk, none of the other three mental health outcomes were significantly predicted. The total indirect effects of positive bystander behavior on mental health variables were also examined: depression (*β* = −0.024, 95% CI = −0.035~−0.014, *p* < 0.05), anxiety (*β* = −0.022, 95% CI = −0.032~−0.012, *p* < 0.05), suicide risk *(β* = −0.018, 95% CI = −0.025~−0.011, *p* < 0.05), and sleep disturbance (*β* = −0.016, 95% CI = −0.024~−0.008, *p* < 0.05). However, all indirect effects of self-efficacy alone were not significant for the four relationships. These findings suggested that coping styles, and self-efficacy partially mediated the relationship between positive bystander behavior and mental health outcomes.

## 4. Discussion

First, in line with hypothesis 1, our study found significant correlations between bullying bystander behavior and its mental health outcomes. The correlation coefficients for positive bystander behavior demonstrated significant negative correlations with suicide risk (−0.025, *p* < 0.001). Conversely, the correlation coefficients for negative bystander behavior demonstrated positive correlations with all four mental health outcomes: anxiety (0.134, *p* < 0.001), depression (0.170, *p* < 0.001), suicide risk (0.164, *p* < 0.001), and sleep disturbance (0.113, *p* < 0.001). The overall results were consistent with previous studies, which indicated the association between witnessing bullying and elevated risk of mental health symptoms: anxiety and social isolation [71], depression [72,73], suicidal intention [40], and psychological distress [74]. Meanwhile, the present study findings were consistent with a part of Evan et al.’s study conducted in the USA [75], which indicated that negative bystander behavior positively correlated with internalizing symptoms. However, the results regarding the association of positive bystanders exhibited opposite directions. The main reason may be attributed to the different samples under distinct cultural backgrounds. Additionally, according to Jason and Hazler [76], simply witnessing bullying can be traumatic in itself. Nonetheless, our findings were more consistent with common sense. Beyond those previous findings, we further extended this result based on the cognitive transaction theory by subdividing bystanders into positive and negative roles within the context of China. We also examined in detail their different relationships with mental health risk factors in comparison with victims and perpetrators.

Second, our research focused on exploring what factors play critical and significant roles, which might explain the mechanism of the relationship between bullying bystander behavior and psychological risk factors. We found that self-efficacy and coping styles not only mediated the associations of bullying victims and perpetrators with mental health risk factors but also significantly mediated the associations between mental health factors and both positive and negative bystanders. Previous work suggested that coping styles mediated the association between bullying perpetration, victimization, witnessing, and suicide risk among adolescents [53]. In addition, research has also identified the mediator role of self-efficacy between victimization and perpetration and mental health in Germany and China [52]. Our results regarding positive bystanders’ defending behaviors were similar to Hutchinson’s study [71], which indicated self-efficacy and moral values contributed to the assertive attitude to hostile peers. According to social cognitive theory and the transaction model of stress and coping, the distinction between coping style and self-efficacy lies in their nature—coping reflects external measures and responses to external stimuli, while self-efficacy involves internal beliefs and realizations within one’s psychological construction system [42,44]. Unlike previous research focusing on only a few risk factors, our mediation study is the first to systematically include the main psychological risk indicators and four bullying behaviors in China.

This study brings critical implications regarding both practical applications and theoretical innovations. On one hand, the results help stakeholders realize that it is important to pay attention to bystanders in the process of public health policy-making and school intervention development. In particular, understanding why bystanders exhibit different trajectories of mental outcomes contributes to identifying protective factors to encourage positive bystander behavior and reduce negative bystander behavior. Such interventions can mitigate the adverse mental health outcomes associated with those behaviors. Thus, enhancing students’ self-efficacy and promoting positive coping styles—particularly in relation to high-scoring items on the specific measurements of these mediators—may reduce the risk of developing anxiety, depression, and ultimately, suicidal ideation. Furthermore, the study prompts us to further reflect on the topic that bullying prevention interventions should be structural and involve a coordinated effort among multiple stakeholders, including lawmakers, law enforcement, parents, the community, and school leaders [47,77]. On the other hand, this study extends theoretical basis by adding analysis of bystander subgroups involving in bullying dynamics. Additionally, this study advances our understanding by integrating self-efficacy and coping styles into the broader framework of bullying dynamics. This theoretical innovation can guide future research in exploring these dynamics in other cultural settings and contribute to more comprehensive models.

Our study is subject to several limitations that warrant discussion. Firstly, the cross-sectional design impedes the ability to establish causality and the direction of relationships. However, longitudinal studies in the past indicated that negative coping strategies contribute to depressive symptoms up to one year later [78], aligning with our mediation model findings. Nevertheless, future longitudinal investigations are crucial for comprehensively understanding these relationships. Secondly, our findings are confined to traditional bullying, overlooking other prevalent forms like cyberbullying. One study suggested potential differences in the relationship between cyberbullying, depressive symptoms, and suicide risk compared to conventional bullying [79]. Exploring various bullying types is essential to understand their impact on mental health indicators. Third, our study solely examined the frequency of bullying victimization and perpetration, without considering crucial aspects like the severity and duration of bullying, which may influence outcomes. Fourth, factors like family functioning, childhood adversity, family history of suicide or mental illness, and peer support, which could contribute to mental health among adolescents, were not addressed in our study. Fifth, due to the questionnaire’s nature, our investigation did not delve into different negative coping styles. Previous studies proposed that bullying victims were more prone to adopt avoidant or emotion-focused coping styles, while non-involved individuals tended to employ problem-focused coping styles. Sixth, The data for this study were collected in a single large, economically developed city in China, which may limit the generalizability of our findings across the country. Future research using nationally representative data will be necessary to extend these results to secondary students in other regions of China. Seventh, the measurements of bullying consisted of single-item questions. Although multi-item measures offer advantages such as improved accuracy and comprehensiveness, evidence suggests that for constructs with clear or specific scopes, single-item measures have not demonstrated significantly worse performance than multi-items [80,81]. Single-item measures are suitable for clear, specific, or objective constructs and for large sample-size studies and time-demanding research, particularly for concepts that can be directly observed or measured [82,83]. Finally, given that 99.7% of the sample was of Han ethnicity, the results cannot be generalized to the broader cultural diversity within China. Existing research from Europe and America showed that belonging to certain minority groups could serve as a protective factor against involvement in bullying [84,85]. It also emphasized the importance of considering ethnic composition when investigating ethnic differences in school bullying [86]. Similarly, a study conducted in China revealed that the dominant ethnic minority, the Yugur, in ethnically diverse regions, exhibited a significantly lower likelihood of being traditionally bullied compared to the national majority, the Han [87]. Future research could further explore ethnic differences in bullying, particularly in bystander behavior.

In conclusion, while the biological mechanisms of the relationship between bullying and mental health symptoms remain unclear, our study identifies different pathways through which bullying behaviors contribute to anxiety, depression, sleep disturbance, and suicide risk. Among these pathways, our study is the first to underscore that coping styles and self-efficacy mediate the relationship between positive and negative bystanders and the four mental symptoms. Given the significance of mental health problems and bullying incidences among adolescents, our findings suggest that having more of a positive coping style and enhanced self-efficacy are critical protective factors for improving mental well-being. These findings can lead the direction for effective prevention strategies and targeted intervention approaches in China.

## Figures and Tables

**Table 1:** Descriptive statistics of participants.

Characteristic	Number of Participants	Percent (%)
Age (Years)		
10–13	164	1.4%
14–17	9936	84.7%
18–21	1634	13.9%
Gender		
Male	6317	53.8%
Female	5417	46.2%
Ethnic group		
Han	11,702	99.7%
Others	32	0.3%
School level		
Junior high school	8741	74.5%
High school	2993	25.5%
School type		
Boarding school	3013	25.7%
Day school	8545	72.8%
Missing	176	1.5%
Residence		
Urban	6735	56.5%
Rural	4399	36.9%
Missing	600	6.6%
Only child in the family		
Yes	4006	33.6%
No	7666	64.3%
Missing	62	2.1%
Father’s education level		
High school or lower	9340	79.6%
College or higher	2385	20.3%
Missing	9	0.1%
Mother’s education level		
High school or lower	9692	82.6%
College or higher	2018	17.2%
Missing	24	0.2%
Bullying victimization		
Never	10,828	92.3%
Sometimes	675	5.8%
Often	144	1.2%
Everyday	87	0.7%
Bullying perpetration		
Never	9820	83.7%
Sometimes	1666	14.2%
Often	178	1.5%
Everyday	70	0.6%
Positive bystander behavior		
None	5599	47.7%
One option	5996	51.1%
Two options	139	1.2%
Negative bystander behavior		
None	10,771	91.8%
One option	959	8.1%
Two options	4	0.1%

**Table 2:** Correlations among the variables.

	BV	BP	Pby	Nby	AS	DS	SR	SD	GESE	Ptcsq	Ntscq
BV	1										
BP	0.268 ***	1									
Pby	0.047 ***	0.044 ***	1								
Nby	0.199 ***	0.200 ***	−0.311 ***	1							
AS	0.135 ***	0.177 ***	0.007	0.134 ***	1						
DS	0.166 ***	0.198 ***	−0.015	0.170 ***	0.815 ***	1					
SR	0.177 ***	0.149 ***	−0.025 ***	0.164 ***	0.415 ***	0.498 ***	1				
SD	0.132 ***	0.161 ***	0.014	0.113 ***	0.477 ***	0.522 ***	0.342 ***	1			
GESE	−0.043 ***	−0.058 ***	0.003	−0.052 ***	−0.218 ***	−0.234 ***	−0.154 ***	−0.157 ***	1		
Ptcsq	−0.083 ***	−0.049 ***	0.037 ***	−0.082 ***	−0.168 ***	−0.186 ***	−0.171 ***	−0.095 ***	0.376 ***	1	
Ntscq	0.087 ***	0.114 ***	−0.043 ***	0.115 ***	0.486 ***	0.510 ***	0.354 ***	0.392 ***	−0.103 ***	0.141 ***	1
*Mean*	0.1	0.2	0.5	0.1	5.2	5.4	2.6	4.3	24.9	30.1	25.5
*SD*	0.4	0.5	0.5	0.3	5.1	6.1	6.2	4.9	6.5	8.4	8.8

Note: *n* = 11,734. BV: Bullying victimization; BP: Bullying perpetration; Nby: Negative bystander behavior; Pby: Positive bystander behavior; Ptcsq: Positive trait coping style; Ntcsq: Negative trait coping style; GSES: General self-efficacy; AS: Anxiety symptom; DS: Depression symptom; SD: Sleep disturbance; SR: Suicide risk; *Mean*: Average of variable; *SD*: Standard deviation; *** *p* < 0.001.

**Table 3:** The standardized coefficients of the mediation model among bullying victimization, coping styles, self-efficacy, and four outcomes.

*n* = 10,713	DS	Ntcsq	Ptcsq	GSES	DS
Sex	0.102 ***	0.136 ***	−0.030 **	−0.146 ***	0.011
Age	0.118 ***	0.117 ***	−0.066 ***	−0.103 ***	0.031 *
School level	0.033	0.041 *	0.037 *	−0.008	0.019
School type	0.032 **	−0.028 *	−0.058 ***	−0.073 ***	0.029 **
Residence	0.005	−0.024 *	−0.029 **	−0.052 ***	0.007
One-child	−0.005	−0.001	0.009	0.003	−0.002
Edu-Father	−0.014	0.001	0.043 ***	0.073 ***	0.001
Edu-Mother	0.005	0.020	0.041 ***	0.080 ***	0.010
BV	0.203 ***	0.092 ***	−0.088 ***	−0.048 ***	0.128 ***
Ntcsq					0.541 ***
Ptcsq					−0.240 ***
GSES					−0.072 ***
R^2^	0.064	0.053	0.021	0.059	0.352
Total indirect effect:	*β*: 0.075	Boot SE: 0.006		Boot 95% CI: [0.064, 0.086]	
Indirect path1: Ntcsq	*β*: 0.050	Boot SE: 0.006		Boot 95% CI: [0.038, 0.063]	
Indirect path2: Ptcsq	*β*: 0.021	Boot SE: 0.003		Boot 95% CI: [0.015, 0.027]	
Indirect path3: GSES	*β*: 0.004	Boot SE: 0.001		Boot 95% CI: [0.002, 0.005]	
***n*** **= 10,713**	**AS**	**Ntcsq**	**Ptcsq**	**GSES**	**AS**
Sex	0.127 ***	0.136 ***	−0.030 **	−0.146 ***	0.041 ***
Age	0.102 ***	0.117 ***	−0.066 ***	−0.103 ***	0.021
School level	0.054 **	0.041 *	0.037 *	−0.008	0.041 **
School type	0.012	−0.028 *	−0.058 ***	−0.073 ***	0.009
Residence	0.012	−0.024 *	−0.029 **	−0.052 ***	0.014
One-child	−0.002	−0.001	0.009	0.003	0.001
Edu-Father	−0.010	0.001	0.043 ***	0.073 ***	0.005
Edu-Mother	−0.002	0.020	0.041 ***	0.080 ***	0.003
BV	0.161 ***	0.092 ***	−0.088 ***	−0.048 ***	0.092 ***
Ntcsq					0.504 ***
Ptcsq					−0.219 ***
GSES					−0.074 ***
R^2^	0.059	0.053	0.021	0.059	0.309
Total indirect effect:	*β*: 0.069	Boot SE: 0.005		Boot 95% CI: [0.059, 0.080]	
Indirect path1: Ntcsq	*β*: 0.047	Boot SE: 0.006		Boot 95% CI: [0.035, 0.058]	
Indirect path2: Ptcsq	*β*: 0.019	Boot SE: 0.003		Boot 95% CI: [0.014, 0.024]	
Indirect path3: GSES	*β*: 0.004	Boot SE: 0.001		Boot 95% CI: [0.002,0.006]	
***n*** **= 10,713**	**SR**	**Ntcsq**	**Ptcsq**	**GSES**	**SR**
Sex	0.101 ***	0.136 ***	−0.030 **	−0.146 ***	0.049 ***
Age	0.037 *	0.117 ***	−0.066 ***	−0.103 ***	−0.016
School level	−0.032	0.041 *	0.037 *	−0.008	−0.038 *
School type	0.050 ***	−0.028 *	−0.058 ***	−0.073 ***	0.048 ***
Residence	−0.013	−0.024 *	−0.029 **	−0.052 ***	−0.011
One-child	0.008	−0.001	0.009	0.003	0.011
Edu-Father	−0.017	0.001	0.043 ***	0.073 ***	−0.008
Edu-Mother	−0.005	0.020	0.041 ***	0.080 ***	−0.004
BV	0.198 ***	0.092 ***	−0.088 ***	−0.048 ***	0.150 ***
Ntcsq					0.337 ***
Ptcsq					−0.196 ***
GSES					−0.004
R^2^	0.050	0.053	0.023	0.059	0.162
Total indirect effect:	*β*: 0.049	Boot SE: 0.003		Boot 95% CI: [0.042, 0.055]	
Indirect path1: Ntcsq	*β*: 0.031	Boot SE: 0.004		Boot 95% CI: [0.023, 0.039]	
Indirect path2: Ptcsq	*β*: 0.017	Boot SE: 0.003		Boot 95% CI: [0.012, 0.022]	
Indirect path3: GSES	*β*: 0.000	Boot SE: 0.001		Boot 95% CI: [−0.001, 0.001]	
***n*** **= 10,713**	**SD**	**Ntcsq**	**Ptcsq**	**GSES**	**SD**
Sex	0.078 ***	0.136 ***	−0.030 **	−0.146 ***	0.010
Age	0.069 ***	0.117 ***	0.066 ***	−0.103 ***	0.008
School level	−0.048 **	0.041 *	0.037 *	−0.008	−0.060 ***
School type	−0.024 *	−0.028 *	−0.058 ***	−0.073 ***	−0.023 *
Residence	0.000	−0.024 *	−0.029 **	−0.052 ***	0.003
One-child	0.004	−0.001	0.009	0.003	0.006
Edu-Father	0.011	0.001	0.043 ***	0.073 ***	0.021
Edu-Mother	−0.007	0.020	0.041 ***	0.080 ***	−0.005
BV	0.161 ***	0.092 ***	−0.088 ***	−0.048 ***	0.110 ***
Ntcsq					0.406 ***
Ptcsq					−0.117 ***
GSES					−0.059 ***
R^2^	0.032	0.053	0.021	0.059	0.187
Total indirect effect:	*β*: 0.057	Boot SE: 0.004		Boot 95% CI: [0.042, 0.059]	
Indirect path1: Ntcsq	*β*: 0.038	Boot SE: 0.005		Boot 95% CI: [0.029, 0.047]	
Indirect path2: Ptcsq	*β*: 0.010	Boot SE: 0.002		Boot 95% CI: [0.007, 0.014]	
Indirect path3: GSES	*β*: 0.003	Boot SE: 0.001		Boot 95% CI: [0.001, 0.005]	

Note: BV: Bullying victimization; Ptcsq: Positive trait coping style; Ntcsq: Negative trait coping style; GSES: General self-efficacy; DS: Depression symptom; AS: Anxiety symptom; SR: Suicide risk; SD: Sleep disturbance; One-child: One child family; Edu-father: Education level of father; Edu-Mother: Education level of mother. * *p* < 0.05, ** *p* < 0.01, *** *p* < 0.001.

**Table 4:** The standardized coefficients of the mediation model among bullying perpetration, coping styles, self-efficacy, and four outcomes.

*n* = 10,713	DS	Ntcsq	Ptcsq	GSES	DS
Sex	0.113 ***	0.146 ***	−0.029 **	−0.150 ***	0.017 ***
Age	0.109 ***	0.112 ***	−0.063 ***	−0.101 ***	0.026 ***
School level	0.033	0.041 *	0.038 *	−0.008	0.019
School type	0.033 **	−0.029 *	−0.059 **	−0.073 ***	0.029 **
Residence	0.006	−0.023 *	−0.030 **	−0.052 ***	0.008
One-child	−0.002	0.001	0.007	0.001	0.001
Edu-Father	−0.010	0.003	0.043 ***	0.072 ***	0.004
Edu-Mother	0.006	0.021	0.040 ***	0.080 ***	0.011
BP	0.199 ***	0.124 ***	−0.047 ***	−0.058 ***	0.116 ***
Ntcsq					0.540 ***
Ptcsq					−0.247 ***
GSES					−0.069 ***
R^2^	0.062	0.059	0.015	0.060	0.349
Total indirect effect:	*β*: 0.082	Boot SE: 0.005		Boot 95% CI: [0.072, 0.093]	
Indirect path1: Ntcsq	*β*: 0.067	Boot SE: 0.006		Boot 95% CI: [0.056, 0.078]	
Indirect path2: Ptcsq	*β*: 0.012	Boot SE: 0.003		Boot 95% CI: [0.007, 0.017]	
Indirect path3: GSES	*β*: 0.004	Boot SE: 0.001		Boot 95% CI: [0.002, 0.006]	
***n*** **= 10,713**	**AS**	**Ntcsq**	**Ptcsq**	**GSES**	**AS**
Sex	0.140 ***	0.146 ***	−0.029 **	−0.150 ***	0.050 ***
Age	0.095 ***	0.112 ***	−0.063 ***	−0.101 ***	0.018
School level	0.055 **	0.041 *	0.038 *	−0.008	0.042 *
School type	0.012	−0.029 *	−0.059 **	−0.073 ***	0.008
Residence	0.013	−0.023 *	−0.030 **	−0.052 ***	0.014
One-child	0.002	0.001	0.007	0.001	0.003
Edu-Father	−0.006	0.003	0.043 ***	0.072 ***	0.007
Edu-Mother	−0.001	0.021	0.040 ***	0.080 ***	0.004
BP	0.185 ***	0.124 ***	−0.047 ***	−0.058 ***	0.108 ***
Ntcsq					0.500 ***
Ptcsq					−0.222 ***
GSES					−0.072 ***
R^2^	0.067	0.059	0.015	0.060	0.312
Total indirect effect:	*β*: 0.076	Boot SE: 0.005		Boot 95% CI: [0.070, 0.086]	
Indirect path1: Ntcsq	*β*: 0.062	Boot SE: 0.005		Boot 95% CI: [0.051, 0.072]	
Indirect path2: Ptcsq	*β*: 0.103	Boot SE: 0.002		Boot 95% CI: [0.006, 0.015]	
Indirect path3: GSES	*β*: 0.004	Boot SE: 0.001		Boot 95% CI: [0.002, 0.006]	
***n*** **= 10,713**	**SR**	**Ntcsq**	**Ptcsq**	**GSES**	**SR**
Sex	0.106 ***	0.146 ***	−0.029 **	−0.150 ***	0.050 ***
Age	0.030	0.112 ***	0.063 ***	−0.101 ***	−0.022
School level	−0.032	0.041 *	0.038 *	−0.008	−0.038 **
School type	0.052 ***	−0.029 *	−0.059 ***	−0.073 ***	0.050 ***
Residence	−0.012	−0.023 *	−0.030 **	−0.052 ***	−0.010
One-child	0.013	0.001	0.007	0.001	0.014
Edu-Father	−0.014	0.003	0.043 ***	0.072 ***	−0.006
Edu-Mother	−0.004	0.021	0.040 ***	0.080 ***	−0.002
BP	0.152 ***	0.124 ***	−0.047 ***	−0.058 ***	0.100 ***
Ntcsq					0.341 ***
Ptcsq					−0.207 ***
GSES					−0.001
R^2^	0.034	0.059	0.015	0.060	0.150
Total indirect effect:	*β*: 0.052	Boot SE: 0.003		Boot 95% CI: [0.045, 0.059]	
Indirect path1: Ntcsq	*β*: 0.042	Boot SE: 0.004		Boot 95% CI: [0.035, 0.050]	
Indirect path2: Ptcsq	*β*: 0.010	Boot SE: 0.002		Boot 95% CI: [0.006, 0.014]	
Indirect path3: GSES	*β*: 0.000	Boot SE: 0.001		Boot 95% CI: [−0.001, 0.002]	
***n*** **= 10,713**	**SD**	**Ntcsq**	**Ptcsq**	**GSES**	**SD**
Sex	0.087 ***	0.146 ***	−0.029 **	−0.150 ***	0.017
Age	0.062 ***	0.112 ***	0.063 ***	−0.101 ***	0.003
School level	−0.047 **	0.041 *	0.038 *	−0.008	−0.060 ***
School type	−0.023 *	−0.029 *	−0.059 ***	−0.073 ***	−0.023 *
Residence	0.001	−0.023 *	−0.030 **	−0.052 ***	0.004
One-child	0.008	0.001	0.007	0.001	0.008
Edu-Father	0.015	0.003	0.043 ***	0.072 ***	0.023 *
Edu-Mother	−0.006	0.021	0.040 ***	0.080 ***	−0.005
BP	0.167 ***	0.124 ***	−0.047 ***	−0.058 ***	0.108 ***
Ntcsq					0.404 ***
Ptcsq					−0.122 ***
GSES					−0.056 ***
R^2^	0.034	0.059	0.015	0.060	0.187
Total indirect effect:	*β*: 0.059	Boot SE: 0.004		Boot 95% CI: [0.051, 0.067]	
Indirect path1: Ntcsq	*β*: 0.050	Boot SE: 0.004		Boot 95% CI: [0.042, 0.058]	
Indirect path2: Ptcsq	*β*: 0.006	Boot SE: 0.001		Boot 95% CI: [0.003, 0.009]	
Indirect path3: GSES	*β*: 0.003	Boot SE: 0.001		Boot 95% CI: [0.002, 0.005]	

Note: BP: Bullying perpetration; Ptcsq: Positive trait coping style; Ntcsq: Negative trait coping style; GSES: General self-efficacy; DS: Depression symptom; AS: Anxiety symptom; SR: Suicide risk; SD: Sleep disturbance; One-child: One child family; Edu-father: Education level of father; Edu-Mother: Education level of mother. * *p* < 0.05, ** *p* < 0.01, *** *p* < 0.001.

**Table 5:** The standardized coefficients of the mediation model among negative bystander behavior, coping styles, self-efficacy, and four outcomes.

*n* = 10,713	DS	Ntcsq	Ptcsq	GSES	DS
Sex	0.103 ***	0.139 ***	−0.031 **	−0.147 ***	0.010
Age	0.108 ***	0.112 ***	0.062 ***	−0.101 ***	0.026
School level	0.023	0.035 *	0.042 *	−0.005	0.014
School type	0.029 **	−0.031 *	−0.056 ***	−0.072 ***	0.027 *
Residence	0.007	−0.022 *	−0.031 **	−0.053 ***	0.008
One-Child	−0.004	−0.001	0.009	0.002	−0.002
Edu-Father	−0.012	−0.002	0.042 ***	0.073 ***	0.003
Edu-Mother	0.001	0.017	0.043 ***	0.082 ***	0.008
Nby	0.192 ***	0.113 ***	−0.086 ***	−0.054 ***	0.107 ***
Ntcsq					0.541 ***
Ptcsq					−0.242 ***
GSES					−0.072 ***
R^2^	0.060	0.057	0.020	0.060	0.347
Total indirect effect:	*β*: 0.086	Boot SE: 0.005		Boot 95% CI: [0.075, 0.096]	
Indirect Path1: Ntcsq	*β*: 0.061	Boot SE: 0.006		Boot 95% CI: [0.050, 0.072]	
Indirect Path2: Ptcsq	*β*: 0.021	Boot SE: 0.003		Boot 95% CI: [0.016, 0.026]	
Indirect Path3: GSES	*β*: 0.004	Boot SE: 0.001		Boot 95% CI: [0.002, 0.006]	
***n*** **= 10,713**	**AS**	**Ntcsq**	**Ptcsq**	**GSES**	**AS**
Sex	0.129 ***	0.139 ***	−0.031 **	−0.147 ***	0.041 ***
Age	0.095 ***	0.112 ***	−0.062 ***	−0.101 ***	0.017
School level	0.047 **	0.035 *	0.042 *	−0.005	0.038 **
School type	0.009	−0.031 *	−0.056 ***	−0.072 ***	0.007
Residence	0.014	−0.022 *	−0.031 **	−0.053 ***	0.015
One-Child	−0.001	−0.001	0.009	0.002	0.001
Edu-Father	−0.008	−0.002	0.042 ***	0.073 ***	0.005
Edu-Mother	−0.005	0.017	0.043 ***	0.082 ***	0.001
Nby	0.157 ***	0.113 ***	−0.086 ***	−0.054 ***	0.078 ***
Ntcsq					0.504 ***
Ptcsq					−0.220 ***
GSES					−0.074 ***
R^2^	0.058	0.057	0.020	0.060	0.307
Total indirect effect:	*β*: 0.080	Boot SE: 0.005		Boot 95% CI: [0.070, 0.090]	
Indirect Path1: Ntcsq	*β*: 0.057	Boot SE: 0.005		Boot 95% CI: [0.047, 0.067]	
Indirect Path2: Ptcsq	*β*: 0.019	Boot SE: 0.003		Boot 95% CI: [0.014, 0.024]	
Indirect Path3: GSES	*β*: 0.004	Boot SE: 0.001		Boot 95% CI: [0.002, 0.006]	
***n*** **= 10,713**	**SR**	**Ntcsq**	**Ptcsq**	**GSES**	**SR**
Sex	0.100 ***	0.139 ***	−0.031 **	−0.147 ***	0.046 ***
Age	0.028 *	0.112 ***	0.062 ***	−0.101 ***	−0.023
School level	−0.040 *	0.035 *	0.042 *	−0.005	−0.044 **
School type	0.048 ***	−0.031 *	−0.056 ***	−0.072 ***	0.047 ***
Residence	−0.010	−0.022 *	−0.031 **	−0.053 ***	−0.009
One-child	−0.015	−0.001	0.009	0.002	0.012
Edu-Father	−0.015	−0.002	0.042 ***	0.073 ***	−0.007
Edu-Mother	−0.009	0.017	0.043 ***	0.082 ***	−0.006
Nby	−0.039 ***	0.113 ***	−0.086 ***	−0.054 ***	0.112 ***
Ntcsq					0.339 ***
Ptcsq					−0.201 ***
GSES					−0.003
R^2^	0.039	0.057	0.020	0.060	0.152
Total indirect effect:	*β*: 0.056	Boot SE: 0.004		Boot 95% CI: [0.049, 0.063]	
Indirect path1: Ntcsq	*β*: 0.038	Boot SE: 0.004		Boot 95% CI: [0.031, 0.046]	
Indirect path2: Ptcsq	*β*: −0.017	Boot SE: 0.002		Boot 95% CI: [0.013, 0.022]	
Indirect path3: GSES	*β*: 0.000	Boot SE: 0.0001		Boot 95% CI: [−0.001, −0.002]	
***n*** **= 10,713**	**SD**	**Ntcsq**	**Ptcsq**	**GSES**	**SD**
Sex	0.076 ***	0.139 ***	−0.031 **	−0.147 ***	0.007
Age	0.063 ***	0.112 ***	0.062 ***	−0.101 ***	0.003
School level	−0.054 **	0.035 *	0.042 *	−0.005	−0.064 ***
School type	−0.025 *	−0.031 *	−0.056 ***	−0.072 ***	−0.024 *
Residence	0.001	−0.022 *	−0.031 **	−0.053 ***	0.004
One-child	0.005	−0.001	0.009	0.002	0.007
Edu-Father	0.013	−0.002	0.042 ***	0.073 ***	0.021
Edu-Mother	−0.009	0.017	0.043 ***	0.082 ***	−0.007
Nby	0.126 ***	0.113 ***	−0.086 ***	−0.054 ***	0.066 ***
Ntcsq					0.410 ***
Ptcsq					−0.122 ***
GSES					−0.059 ***
R^2^	0.022	0.057	0.020	0.060	0.180
Total indirect effect:	*β*: 0.060	Boot SE: 0.004		Boot 95% CI: [0.052, 0.068]	
Indirect path1: Ntcsq	*β*: 0.046	Boot SE: 0.004		Boot 95% CI: [0.038, 0.055]	
Indirect path2: Ptcsq	*β*: 0.011	Boot SE: 0.002		Boot 95% CI: [0.008, 0.014]	
Indirect path3: GSES	*β*: 0.003	Boot SE: 0.001		Boot 95% CI: [0.002, 0.005]	

Note: Nby: Negative bystander behavior; Ptcsq: Positive trait coping style; Ntcsq: Negative trait coping style; GSES: General self-efficacy; DS: Depression symptom; AS: Anxiety symptom; SR: Suicide risk; SD: Sleep disturbance; One-child: One child family; Edu-father: Education level of father; Edu-Mother: Education level of mother. * *p* < 0.05, ** *p* < 0.01, *** *p* < 0.001.

**Table 6:** The standardized coefficients of the mediation model among positive bystander behavior, coping styles, self-efficacy, and four outcomes.

*n* = 10,713	DS	Ntcsq	Ptcsq	GSES	DS
Sex	0.087 ***	0.130 ***	−0.024 *	−0.143 ***	−0.002
Age	0.115 ***	0.115 ***	0.064 ***	−0.103 ***	0.027
School level	0.031	0.038 *	0.041 *	−0.008	0.019
School type	0.038 ***	−0.025 *	−0.061 ***	−0.075 ***	0.032 ***
Residence	0.004	−0.025 *	−0.029 **	−0.052 ***	0.006
One-child	−0.003	−0.0002	0.008	0.002	−0.001
Edu-Father	−0.016	−0.0002	0.044 ***	0.074 ***	0.001
Edu-Mother	0.006	0.020	0.415 ***	0.080 ***	0.011
Pby	−0.011	−0.026 **	0.039 ***	−0.007	0.013
Ntcsq					0.558 ***
Ptcsq					−0.256 ***
GSES					−0.072 ***
R^2^	0.024	0.045	0.014	0.057	0.336
Total indirect effect:	*β*: −0.024	Boot SE: 0.005		Boot 95% CI: [−0.035, −0.014]	
Indirect path1: Ntcsq	*β*: −0.015	Boot SE: 0.005		Boot 95% CI: [−0.025, −0.005]	
Indirect path2: Ptcsq	*β*: −0.010	Boot SE: 0.003		Boot 95% CI: [−0.015, −0.005]	
Indirect path3: GSES	*β*: 0.001	Boot SE: 0.001		Boot 95% CI: [−0.001, 0.002]	
***n*** **= 10,713**	**AS**	**Ntcsq**	**Ptcsq**	**GSES**	**AS**
Sex	0.115 ***	0.130 ***	−0.024 **	−0.143 ***	0.032 ***
Age	0.10 ***	0.115 ***	−0.064 ***	−0.103 ***	0.018
School level	0.055 **	0.038 *	0.041 *	−0.008	0.044 **
School type	0.017	−0.025 *	−0.061 ***	−0.075 ***	0.011
Residence	0.000	−0.025 *	−0.029 **	−0.052 ***	0.013
One-Child	0.011	−0.0002	0.008	0.002	0.002
Edu-Father	−0.011	−0.0002	0.044 ***	0.074 ***	0.004
Edu-Mother	−0.001	0.0196	0.042 ***	0.080 ***	0.005
Pby	0.013	−0.026 **	0.039 ***	−0.007	0.035 ***
Ntcsq					0.517 ***
Ptcsq					−0.232 ***
GSES					−0.073 ***
R^2^	0.034	0.045	0.014	0.057	0.302
Total indirect effect:	*β*: −0.022	Boot SE: 0.005		Boot 95% CI: [−0.032, −0.012]	
Indirect Path1: Ntcsq	*β*: −0.014	Boot SE: 0.005		Boot 95% CI: [−0.023, −0.004]	
Indirect Path2: Ptcsq	*β*: −0.009	Boot SE: 0.003		Boot 95% CI: [−0.013, −0.005]	
Indirect Path3: GSES	*β*: 0.001	Boot SE: 0.001		Boot 95% CI: [−0.001, 0.002]	
***n*** **= 10,713**	**SR**	**Ntcsq**	**Ptcsq**	**GSES**	**SR**
Sex	0.087 ***	0.130 ***	−0.024 *	−0.143 ***	0.035 ***
Age	0.033 *	0.115 ***	0.064 ***	−0.103 ***	−0.022
School level	−0.036 *	0.038 *	0.041 *	−0.008	−0.041 **
School type	0.056 ***	−0.025 *	−0.061 ***	−0.075 ***	0.052 ***
Residence	0.010	−0.025 *	−0.029 **	−0.052 ***	−0.011
One-child	−0.014	−0.0002	0.008	0.002	0.012
Edu-Father	−0.018	−0.0002	0.044 ***	0.074 ***	−0.009
Edu-Mother	−0.005	0.020	0.415 ***	0.080 ***	−0.003
Pby	−0.039 ***	−0.026 **	0.039 ***	−0.007	−0.022 *
Ntcsq					0.355 ***
Ptcsq					−0.213 ***
GSES					−0.004
R^2^	0.013	0.045	0.014	0.057	0.141
Total indirect effect:	*β*: −0.018	Boot SE: 0.004		Boot 95% CI: [−0.025, −0.011]	
Indirect path1: Ntcsq	*β*: −0.009	Boot SE: 0.003		Boot 95% CI: [−0.016, −0.031]	
Indirect path2: Ptcsq	*β*: −0.008	Boot SE: 0.002		Boot 95% CI: [−0.013, −0.004]	
Indirect path3: GSES	*β*: 0.000	Boot SE: 0.0001		Boot 95% CI: [−0.0003, 0.0004]	
***n*** **= 10,713**	**SD**	**Ntcsq**	**Ptcsq**	**GSES**	**SD**
Sex	0.066 ***	0.130 ***	−0.024 *	−0.143 ***	−0.001
Age	0.067 ***	0.115 ***	0.064 ***	−0.103 ***	0.004
School level	−0.049 **	0.038 *	0.041 *	−0.008	−0.060 ***
School type	−0.019 ***	−0.025 *	−0.061 ***	−0.075 ***	0.021 *
Residence	0.006	−0.025 *	−0.029 **	−0.052 ***	0.002
One-child	−0.001	−0.0002	0.008	0.002	0.007
Edu-Father	0.010	−0.0002	0.044 ***	0.074 ***	0.020
Edu-Mother	−0.006	0.020	0.415 ***	0.080 ***	−0.004
Pby	−0.002	−0.026 **	0.039 ***	−0.007	0.014
Ntcsq					0.421 ***
Ptcsq					−0.131 ***
GSES					−0.059 ***
R^2^	0.006	0.045	0.014	0.057	0.176
Total indirect effect:	*β*: −0.016	Boot SE: 0.004		Boot 95% CI: [−0.024, −0.008]	
Indirect path1: Ntcsq	*β*: −0.111	Boot SE: 0.004		Boot 95% CI: [−0.019, −0.003]	
Indirect path2: Ptcsq	*β*: −0.005	Boot SE: 0.001		Boot 95% CI: [−0.008, −0.003]	
Indirect path3: GSES	*β*: 0.0004	Boot SE: 0.001		Boot 95% CI: [−0.001, 0.002]	

Note: Pby: Positive bystander behavior; Ptcsq: Positive trait coping style; Ntcsq: Negative trait coping style; GSES: General self-efficacy; DS: Depression symptom; AS: Anxiety symptom; SR: Suicide risk; SD: Sleep disturbance; One-child: One child family; Edu-father: Education level of father; Edu-Mother: Education level of mother. * *p* < 0.05, ** *p* < 0.01, *** *p* < 0.001.

## Data Availability

The data that support the findings of this study are available from the corresponding author upon reasonable request.
